# Cefepime failure for the treatment of cefepime-susceptible, KPC-producing *Escherichia coli* emphysematous cystitis

**DOI:** 10.1128/asmcr.00094-25

**Published:** 2025-07-31

**Authors:** Jordynne Morelan, Rong Hou, Robin Chamberland, Christian M. Gill

**Affiliations:** 1 Department of Pharmacy, SSM Health Saint Louis University Hospital 25213, St. Louis, Missouri, USA; 2 Department of Internal Medicine, Saint Louis University 7547, St. Louis, Missouri, USA; 3 Department of Pathology, Saint Louis University 7547, St. Louis, Missouri, USA; 4 Center for Anti-Infective Research and Development, Hartford Hospital 23893, Hartford, Connecticut, USA; Rush University Medical Center, Chicago, Illinois, USA

**Keywords:** KPC, molecular diagnostics, urinary tract infections

## Abstract

**Background:**

The growing incidence of carbapenem-resistant Enterobacterales (CRE) poses a challenge to clinical practice. Despite some *Klebsiella pneumoniae* carbapenemase (KPC)-positive Enterobacterales testing as susceptible to cefepime, *in vivo* data show that cefepime is not a reliable treatment option for infections caused by carbapenemase-producing CRE (CP-CRE). Contemporary guidance recommends against cefepime use for the treatment of KPC-positive infections; however, the clinical implications of this practice are unknown.

**Case Summary:**

Here, we present a case of clinical failure after cefepime treatment for a KPC-producing *Escherichia coli* urinary tract infection that tested as cefepime-susceptible *in vitro* that was successfully treated with ceftazidime/avibactam. This case is concordant with the *in vivo* PKPD findings that the presence of KPC blunted the microbiologic activity of human-simulated cefepime exposures despite MICs that would suggest susceptible or susceptible dose-dependent (SDD) interpretation.

**Conclusion:**

This case adds to the growing literature supporting the use of carbapenemase testing for Enterobacterales isolates that demonstrate resistance to one or more carbapenems to guide therapy. Until confirmatory clinical data are available, clinicians and laboratorians must consider selective reporting or other strategies in the setting of contradictory phenotypic susceptibility data and genotypic resistance markers.

## INTRODUCTION

Carbapenem-resistant Enterobacterales (CRE) are a growing global public health concern, and detection of these organisms increases along with associated increases in morbidity and mortality (1). Among CRE, carbapenemase-producing isolates (CP-CRE) are notably challenging as such mechanisms confer resistance to most commonly utilized β-lactam agents (2). In the United States, *Klebsiella pneumoniae* carbapenemase (KPC)-producing organisms make up the majority of CP-CRE encountered clinically (3, 4). Indeed, some CP-CRE isolates report as susceptible or susceptible dose-dependent (SDD) to cefepime per Clinical and Laboratory Standards Institute (CLSI) standards (5). In particular, isolates harboring KPC have been reported as cefepime-susceptible (MIC ≤2 mg/L) or SDD (MIC 4 or 8 mg/L) despite cefepime being a known substrate for KPC (5). *In vivo* analyses have demonstrated that 1-log bacterial reduction is unattainable with cefepime in both CP-CRE harboring KPC and non-CP-CRE. In these analyses, the observed efficacy was lower in the CP-CRE isolates, as was demonstrated by significantly less bacterial killing among CP-CRE isolates compared with non-CP-CRE isolates despite the isolates having similar cefepime MICs and treatment with the same human simulated exposures of cefepime 2 g IV every 8 h (6).

In response to this increase in CP-CRE isolates and variations in susceptibility reporting, CLSI has recommended carbapenemase testing using phenotypic and/or molecular assays for any isolates demonstrating resistance to any carbapenem tested (7). Ideally, assays performed should detect and differentiate between specific carbapenemase types (i.e., IMP, KPC, NDM, OXA-48, VIM). The Infectious Diseases Society of America (IDSA) echoes this recommendation and strongly encourages testing for specific carbapenemases produced by clinical CRE isolates to guide antimicrobial therapy (3). Herein, we describe a case of clinical failure when cefepime was used for the treatment of a KPC-producing *Escherichia coli* urinary tract infection that tested as cefepime-susceptible *in vitro*.

## CASE PRESENTATION

A 67-year-old male with a past medical history of alcohol abuse, hepatitis C, cirrhosis, type 2 diabetes, cholangiocarcinoma, end-stage renal disease on intermittent hemodialysis, benign prostatic hyperplasia with prior transurethral prostate resection, and an extensive history of urinary tract infections with multi-drug-resistant organisms was admitted for worsening altered mental status during hemodialysis. A urinalysis performed on hospital day 1 demonstrated pyuria (WBC >100 cells/hpf), 3+ leukocyte esterase, and 3+ bacteria. This urinalysis was consistent with previous urine studies and was reflexed to culture. The patient was initiated on empiric cefepime 1 g IV every 24 h (renally dosed) based on previous culture result of cefepime and meropenem-susceptible *Escherichia coli*, and an infectious diseases consultation was placed for clinical guidance. Despite a lack of typical urinary tract infection (UTI) symptoms (urinary urgency, suprapubic pain, etc.), the infectious diseases consult service decided to treat the presumed UTI based on a documented history of altered mental status with prior UTIs.

On hospital day 4, urine culture results confirmed >100,000 CFU/mL *Escherichia coli* with reported susceptibility to cefepime (MIC <1 mg/L) although this isolate was tested as meropenem-resistant (Table 1). Clinical improvement was observed, so cefepime was continued for a total duration of 9 days per recommendation by infectious diseases consultation, and the patient was discharged on hospital day 13. Per local standards, the isolate was submitted to the state public health laboratory for carbapenemase testing where they determined after discharge (day 22) that the isolate was phenotypically carbapenemase-positive by modified carbapenem inactivation method and KPC-positive by PCR (7). At this time, the patient had received a full course of cefepime and had been discharged following clinical improvement, so no antimicrobial modification was feasible.

**TABLE 1:** Antimicrobial susceptibility testing results for each *Escherichia coli* isolate^*a*^

	Isolate 1 Day 1		Isolate 2 Day 22	
	MIC	STIC	MIC	STIC
Amikacin	4 mg/L	Susceptible	16 mg/L	Resistant
Ampicillin/sulbactam	≥32 mg/L	Resistant	≥32 mg/L	Resistant
Cefazolin	≥64 mg/L	Resistant	≥64 mg/L	Resistant
Cefazolin (urine)	≥64 mg/L	Resistant	≥64 mg/L	Resistant
Cefepime	≤1 mg/L	Susceptible	8 mg/L	SDD
Ceftazidime/avibactam	0.25 mg/L	Susceptible	0.5 mg/L	Susceptible
Ceftriaxone	8 mg/L	Resistant	≥64 mg/L	Resistant
Ciprofloxacin	≥4 mg/L	Resistant	≥4 mg/L	Resistant
Fosfomycin	Susceptible		Susceptible	
Gentamicin	≤1 mg/L	Susceptible	≤1 mg/L	Susceptible
Meropenem	≥16 mg/L	Resistant	≥16 mg/L	Resistant
Meropenem/vaborbactam	NA	NA	0.032 mg/L	Susceptible
Piperacillin/tazobactam	≥128 mg/L	Resistant	≥128 mg/L	Resistant
Tobramycin	8 mg/L	Resistant	≥16 mg/L	Resistant
Trimethoprim/sulfamethoxazole	≥320 mg/L	Resistant	≥320 mg/L	Resistant
KPC gene	Detected		NA	

^*a*^ STIC, susceptibility testing interpretive criteria; SDD, susceptible-dose dependent; NA, not available. Susceptibility testing was performed using Etests (bioMerieux, Durham, NC, USA) for ceftazidime/avibactam and meropenem/vaborbactam. Fosfomycin susceptibility testing was performed using disk diffusion using fosfomycin 200 µg disks (BD BBL, Franklin Lakes, NJ, USA). Vitek 2 Gram-Negative Susceptibility Card AST-GN79 (bioMerieux, Durham, NC, USA) was used for all other susceptibility testing. STIC were determined per CLSI standards.

Nine days after discharge (day 22), the patient returned to the emergency department complaining of 4 days of altered mental status, nausea, vomiting, and diarrhea. At this time, the patient was alert and oriented only to himself (A&O × 1), a decline from his baseline mental status of A&O × 3. It was noted that the patient still had 200 to 300 mL urine output daily. As part of infectious etiology workup, CT scan of the abdomen and pelvis with intravenous contrast was performed, showing extensive gas in the bladder wall with suggestion of possible small foci of gas in the extraperitoneal space suggestive of emphysematous cystitis. CT scan also revealed prostatomegaly measuring 4.6 cm in the transverse dimension. A urinalysis was completed and again demonstrated pyuria (WBC >100 cells/hpf), 2+ leukocyte esterase, and 3+ bacteria. He was subsequently admitted to the internal medicine service for further management.

Ceftazidime/avibactam 0.94 g IV every 24 h (renally dosed) was initiated based on phenotypic and genotypic results from the previous admission (isolate 1) and concern for treatment failure with cefepime. A urine culture (isolate 2) collected on the first day of re-admission (day 22 total) grew *Escherichia coli*, and on day 24, susceptibility results revealed the isolate was cefepime SDD (MIC = 8 mg/L) and exhibited repeated resistance to meropenem (Table 1). Carbapenemase testing was not conducted on isolate 2, though it was putatively considered KPC-positive due to the restoration of meropenem activity in the presence of vaborbactam (4). Ceftazidime/avibactam was continued for a total duration of 14 days to treat the patient’s complicated urinary tract infection. The patient improved clinically and was discharged home on hospital day 15 (day 37 total). No recurrences were observed after ceftazidime/avibactam therapy. The patient was re-admitted for a gastrointestinal bleed on day 47, and a urine culture at that time did not grow *Escherichia coli*.

## DISCUSSION

Though the isolate discussed in this patient case was indeed a KPC-producing *Escherichia coli*, this resistance pattern had not been reported in previous urine cultures despite recurrent UTIs. This lack of information in addition to the notable clinical improvement with cefepime during previous admissions prevented suspicion of underlying resistance. In addition, previous UTIs growing ESBL *Escherichia coli* and *Enterococcus faecium* had been treated with meropenem and vancomycin for seven days. Exposure to carbapenems is a well-documented risk factor for KPC colonization and/or infection (8, 9); however, the influence of previous antimicrobial exposures on the isolation of KPC-positive organisms that remain susceptible to cefepime is unknown.

The advent of novel β-lactam/β-lactamase inhibitors provided much-needed treatment options for infections caused by CP-CRE. Through inhibition of these bacterial resistance mechanisms, these β-lactamase inhibitors allow established agents to perform their antimicrobial activity in isolates that would otherwise be resistant to the agent. For KPC-positive, CP-CRE, ceftazidime/avibactam and meropenem/vaborbactam exhibit potent *in vitro* activity. Data of 187 isolates of KPC-producing Enterobacterales showed that 99.5% were susceptible to ceftazidime/avibactam, and 97.9% were susceptible to meropenem/vaborbactam (4). Avibactam restores activity of ceftazidime by inhibiting activity of Ambler class A and C β-lactamases and some Ambler class D enzymes. Similarly, vaborbactam restores activity of meropenem by inhibiting Ambler class A and C β-lactamases, but it lacks activity against Ambler classes B and D. The expanded activity of these antimicrobials allows for their use in a variety of resistant isolates. The potent *in vitro* activity of the aforementioned β-lactam/β-lactamase inhibitor combinations translated to improved clinical cure and decreased mortality in patients with CRE infections compared with treatment with previous standards of care (i.e., polymyxins) (3, 10, 11).

Although ceftazidime/avibactam and meropenem/vaborbactam also provide reliable activity against less-resistant organisms, they should be reserved for cases where isolates demonstrate resistance to carbapenems in an effort to limit exposure to broad-spectrum antimicrobials. Implementation of rapid diagnostics to identify specific carbapenemases can provide actionable data sooner to help guide therapy to these agents to (i) decrease time to active antimicrobial therapy and (ii) reduce unnecessary use of these agents. Indeed, observational studies have found rapid molecular detection of KPC reduced time to active therapy (3, 10). The benefit of such technology will help build therapeutic algorithms to initiate the most likely active therapy for each carbapenemase class per IDSA Guidance recommendations.

### Conclusion

Here, we describe a case of cefepime treatment failure for a KPC-producing *Escherichia coli* despite *in vitro* susceptibility. This case is concordant with the *in vivo* PKPD findings that the presence of KPC blunted the microbiologic activity of human-simulated cefepime exposures despite MICs that would suggest susceptible or SDD interpretation (6). Similarly, this adds to the growing literature supporting the use of carbapenemase testing in Enterobacterales isolates that demonstrate resistance to one or more carbapenems to guide therapy (3, 7). Together, this raises a patient safety concern for using cefepime for isolates that are KPC-positive, even if susceptible *in vitro*. A systematic assessment of clinical outcomes comparing patients treated with cefepime compared with the novel β-lactams/β-lactamase inhibitors is warranted. Until confirmatory clinical data are available, clinicians and laboratorians must consider selective reporting or other strategies in the setting of contradictory phenotypic susceptibility data and genotypic resistance markers (12, 13).
